# Integrating spatial analysis and questionnaire survey to better understand human-onager conflict in Southern Iran

**DOI:** 10.1038/s41598-021-91921-w

**Published:** 2021-06-14

**Authors:** Alireza Mohammadi, Kamran Almasieh, Ho Yi Wan, Danial Nayeri, Amir Alambeigi, Jason I. Ransom, Samuel A. Cushman

**Affiliations:** 1 Department of Environmental Science and Engineering, Faculty of Natural Resources, University of Jiroft, Jiroft, Iran; 2 Department of Nature Engineering, Agricultural Sciences and Natural Resources University of Khuzestan, Mollasani, Iran; 3 Department of Wildlife, Humboldt State University, 1 Harpst Street, Arcata, CA 95521 USA; 4 Department of Agricultural Extension and Education, College of Agricultural Economics and Development, University of Tehran, Karaj, Iran; 5 Department of Ecosystem Science and Sustainability, Colorado State University, Fort Collins, CO USA; 6 USDA Forest Service Rocky Mountain Research Station, 2500 S. Pine Knoll, Flagstaff, AZ USA

**Keywords:** Biodiversity, Conservation biology, Ecological modelling

## Abstract

Southern Iran is a conservation priority area for the endangered Persian onager (*Equus hemionus onager*), which is threatened by habitat fragmentation and conflict with local communities. To better understand factors that influence onager conservation, we administered a questionnaire in local communities to survey their ecological knowledge, personal experience related to onager, and attitudes toward traditional solutions for reducing crop damage by onager. In addition, we used resistant kernel and factorial least-cost path analyses to identify core areas and corridors for onager movement, and spatial randomization of vehicle collisions and crossing locations to test the predictive ability of resistant kernel and factorial least-cost path predictions of movement. We found that local communities that were knowledgeable about onagers experienced less crop damage from onager compared with those who used traditional methods. Habitat connectivity models revealed that core areas of movement are highly concentrated at the center of protected areas. Some sections of core areas have been cut off by roads where most vehicle collisions with onagers occurred. We propose that effective onager conservation will require integrated landscape-level management to reduce mortality risk, protection of core areas and corridors, development of mitigation strategies to reduce vehicle collisions, and conflict mediation between local communities and onagers.

## Introduction

The Persian Onager (*Equus hemionus onager*) is the only representative of order Perissodactyla in Iran. It is listed as endangered (EN) under the IUCN Red List and is considered the rarest living subspecies of Asiatic wild ass^1,2^. Onager populations in Iran are concentrated in two geographically separate areas: Bahram-e-Goor Protected Area and Touran Protected Area, located in the south and the central parts of Iran, respectively.

In Bahram-e-Goor Protected Area, population estimates in recent years suggest a positive trend^2^. However, there is concern that rising onager abundance may lead to vegetation degradation and water depletion problems^3,4^. Major threats to onager are conflicts resulting from crop-raiding, and road collisions occurring when onager cross roads to access crops^3,5^, and grazing competition between herbivores and livestock^6,7^. Among these threats, agricultural crop-raiding is considered the most important conflict between human and onagers. High crop damage is associated with antagonism of local communities towards this species.

The study region is an arid environment where vegetation is unproductive and sparsely distributed^5^. During the dry summer (from June to August), there is little to no growth in most annual and perennial plants, and herbivores are subject to nutritional stress. Water is a major limiting resource for the onager in this season^5,8,9^. Increases in local onager abundance following better protection measures and management has resulted in onagers moving to agricultural lands which abut the protected areas in which their populations are concentrated during dry months. Such seasonal movement, coupled with an increasing onager population, results in intensified conflicts with the agro-pastoral community such as increasing crop-raiding incidences. Resolving these conflicts is a challenging task for wildlife and land managers^10^. Vehicle collisions pose another substantial threat to the onager population in Bahram-e-Goor Protected Area^3^. The Hassan Abad-Meshkaan road, which skirts the periphery of the area, has impacted the connectivity of the onager population and has become a source of mortality and hazard for both onagers and local communities.

Connectivity models provide practical tools for assessing potential fragmentation effects of roads on wildlife and can help and inform managers for management and conservation planning^11,12^. A wide variety of methods have been proposed for connectivity analysis, including least-cost path modelling^13^, current flow^14^, factorial least-cost path density^15^, resistant kernels^16^ and randomized shortest path algorithm^17^. The factorial least-cost path and cumulative resistant kernel approaches are particularly valuable when used in combination to accurately identify core habitats, fracture zones and corridors across a broad landscape^18,19^.

To support effective conservation and management of the onager population, this study focuses on understanding the drivers of, and mitigation options for, human-onager conflicts. Furthermore, identifying core habitats and movement corridors is essential to identify the locations of the road onager crossing concentrations and to develop comprehensive landscape-scale onager conservation plans. We hypothesized that (1) local people that were more knowledgeable about the ecological role of onagers experience less crop damage compared with those who are less knowledgeable and use traditional methods. (2) Roads are a major driver of human-onager conflicts because they coincide with core areas and corridors used by onagers.

## Materials and methods

### Study area

**Figure 1:**
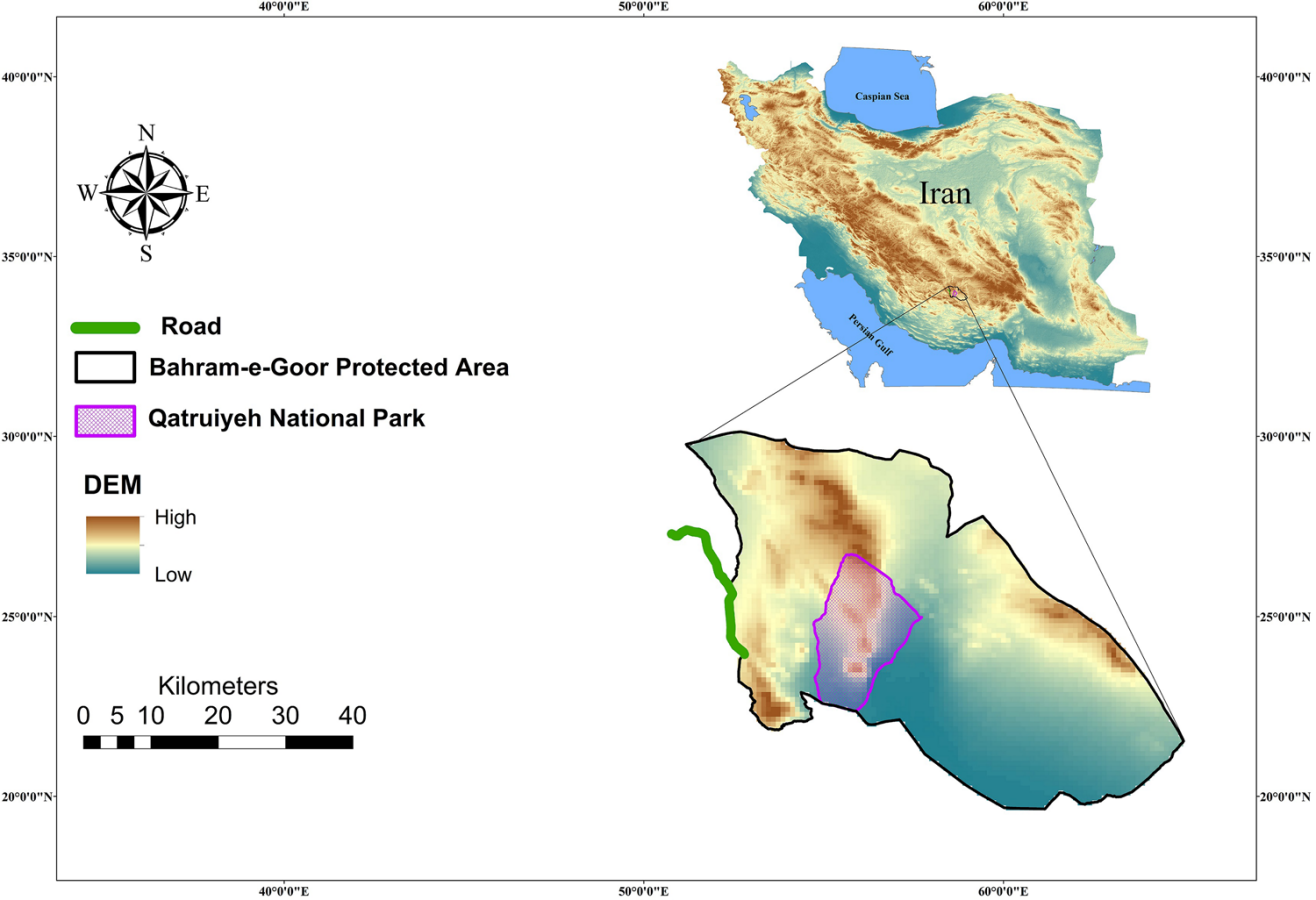
Location of the study area. The software ArcGIS. Version 10.2. was used to generate figure. DEM map was downloaded from the WorldClim database (http://www.worldclim.org).

Qatruiyeh National Park, established in 2008, is a core zone in the Bahram-e-Goor Protected Area (established in 1972) at the border of Fars and Kerman provinces in southern Iran (Fig. 1). It covers 310 km^2^ and is part of the Zagros Mountains. It is a semi-desert with temperate arid climate, vegetated mainly with *Zygophyllum eurypterum* and *Artemisia sieberi*^20^. There are seven villages in the vicinity of the protected area, where pastoralism is the main source of livelihood^21^.

One of the major threats for the Persian onager populations in this area is increasing construction of new roads and increasing road traffic. The Sirjan-Yazd (Hassan Abad-Meshkaan) asphalt road, which passes through the Bahram-e-Goor Protected Area, was recently converted into a highway and represents a substantial threat to Onagers (Fig. 1). This road has two lanes in each direction. The day-time speed limit on this road is 110 km/h and 90 km/h at night. Most vehicles on this road are heavy trucks, which pass at high speed (more than 90 km/h), with high traffic volumes at night. During winter, late autumn and summer of drought years, when fodder is scarce, onagers frequently cross the road to access gardens and agricultural fields, which causes high onager mortality due to vehicle collisions. In this research, we used spatial randomization of vehicle collisions and crossing locations to test the predictive ability of resistant kernel and factorial least-cost path predictions of movement^18^. We also conducted questionnaires with residents from local communities to determine the most important factors influencing human-onager conflicts in the Bahram-e-Goor Protected Area.

### Human-onager conflict assessment

#### Qualitative data collection

We administered a questionnaire through a personal interview to 200 randomly chosen farmers residing near onager populations in the Bahram-e-Goor Protected Area in Fars province. Data were collected through a questionnaire between May and August 2018 (Table S1). Ethical clearance was obtained from the DOE (under permit 32–239). All participants were given a printed descriptive summary of the research (if participants were illiterate, the document was read to them). Prior informed consent was obtained orally from all participants. In this research, we followed legal requirements of ethical issues.

We calculated the sample size needed by using the family size in rural areas around Bahram-e-Goor Protected Area using the Daniel method^22^ (Table S1) as described below (Eq. 1):

We randomly conducted 200 questionnaires in total.

1 $$N=\frac{ {Z}^{2 }P (1-P) }{{d}^{2}}$$

In this equation, *Z* is the Z statistic for a level of confidence, *P* is expected prevalence or proportion (if the expected prevalence is 20%, then P = 0.2), and *d* is precision (if the precision is 5%, then d = 0.05). In this research, we used *d* = 0.5 and *p* was selected according to family sizes in each district of rural areas^22^.

All interviewees were adult males. We collected information on interviewees’ demographic and socioeconomic background (occupation, property, age, and income) as well as their knowledge and opinion on how to prevent onager crop-raiding.

We used logistic regression to analyze the significance of sociological factors related to crop damage by onagers. Our dependent variable was “Have you had any of your crop raided by onager during the last year? (Binary response: 1 = Yes, 0 = No)”. Our independent variables included: (1) traditional solutions for reducing Persian onager damages (Response: 1 guarding dogs, 2: fencing around agricultural land, 3: use of traditional barriers (a plastic cuff with a bell on it), 4: scarecrow, 5: turn on the lights at night , 6: Bird-Scarer (Kalaghparan in Persian); (2) which of these solutions could be effective in reducing Persian onager damages (Responses included: 1: fencing around Persian onager habitat, 2: fencing around farmland, 3: give fodder and provide water for Persian onager, 4: buying fodder from local people by DoE, 4: capturing and relocating Persian onager); (3): do you agree with Persian onager hunting? (Binary response: 1 = Yes, 0= No); (4): what is the role of the Persian onager in the wild? (Response 1: distributing seed of plants, the rangelands are restored, 2: it attracts tourists in the region, 3: beauty of nature: God's creature with a right to live (Intrinsic value), 4: none) (5): age (response: 1: < 30 Years, 2: 30–50 Years, 3: > 50 Years), (6): education (response: 1: Incomplete Elementary (lower than 5th grade of elementary), 2: Complete Elementary (5th grade of elementary), 3: Incomplete High school, 4: Associate Degree, 5: Bachelor of Science (BSc), 5: Master of Science (MSc) or Higher), (7) Experience of Persian onager observation in nature: Have you ever seen a Persian onager in the wild? (Response scale: 1 = Yes, frequently, 2 = Yes, several times, 3: Yes, a few times 4: No, never, 5: only seen the Asiatic wild ass carcass), (8) the presence of a Persian onager around your village damages your farms and gardens. How do you feel about this statement? (Response scale) 1: completely disagree, 2: Somewhat disagree, 3: I do not agree or disagree, 4: I agree somewhat, 5: completely agree.

All statistical tests were conducted in IBM SPSS Statistics (V. 23.0). Independent variables in the logistic regression analysis were coded as showed in Table S1.

### Naïve Bayes classification

Naïve Bayes Classification uses a group of simple classifiers based on probabilities, which are applicable to the types of random independent variables in our study. This approach is a supervised machine learning algorithm based on the Bayes Theorem that is used to solve classification problems by following a probabilistic approach. We used the e1071 library^23^ in R version 3.5.3^24^ for Naïve Bayes classification of onager crop-raiding under this scheme. We considered: Yes (local communities with experience of crop-raid damages), or No (local communities without experience of crop-raid damages during the last one year) as a dependent variable, as a function of the independent variables described in logistic regression section, except we also included farm land area (1: < 1 ha, 2: 1–5 ha, > 5 ha) as an additional variable.

We categorized data into two groups (testing and training) to determine whether the model performed correctly based on training data. Subsequently, 70% of the data were used to test and run the model along with training confirmation. The Naïve Bayes Classifier was trained to anticipate each attitude in the test data. We calculated the randomness of our results using the Mclust library^25^ in R version 3.5.3^24^.

### Onager vehicle collisions

A 25-km section of the 99-km Hassan Abad-Meshkaan road (the area with the highest wildlife-vehicle collision reports) was monitored by motorcycling and walking daily from August to October 2017 (3 weeks). Every morning, we inspected for mammal roadkill within a 30-m buffer on each side of the road, and all carcasses of mammals were recorded using a handheld GPS (Garmin GPS Map 62S). To avoid double-counting, we removed the carcasses after recording. We also obtained collision location data during 2004–2018 from the DoE.

The crossing data for onager were obtained from a variety of sources including opportunistic direct observation, environmental guard’s information, and monitoring by LED portable flashlight at night (summer and autumn seasons of 2017 and 2018).

### Habitat connectivity analysis

#### Habitat suitability modeling

A total of 103 presence points were obtained from DoE (2015) in the study area, including Bahram-e-Goor Protected Area, as well as nearby surroundings. To minimize spatial autocorrelation, a 1-km radius was used to eliminate points around each presence location using the SDM toolbox^26^. The remaining 90 presence points were used in the modeling.

A habitat suitability map for onager was developed using MaxEnt software version 3.3.3k^27^ to create a resistance map for connectivity modeling^28^. We used 10,000 pseudo-absence points^29^. For the training data set, 75% of the presence points were randomly chosen to train and the remaining 25% were used to test the model^30^. We used the area under the ROC curve (AUC) to evaluate model performance. MaxEnt models were completed with 10 bootstrapped replicates.

Environmental layers included in MaxEnt modeling included (1) elevation (digital elevation model [DEM]), (2) slope, (3) land cover, (4) distance from agricultural lands, (5) distance from roads and (6) distance from villages. All layers had a 30 m × 30 m resolution (Table 1).

**Table 1:** Environmental variables used for habitat modeling of the Persian onager in the study area.

Category	Variables	Unit	Source
Topography	Elevation	Meter	https://glovis.usgs.gov
	Slope	Percentage	Elevation
Food and cover	Land-cover	Class	FRWMO, 2010
	Distance from agricultural lands	Degree	Land-cover
Human disturbance	Distance from road	Degree	DoE, 2018
	Distance from villages	Degree	DoE, 2018

Slope was calculated from the DEM layer. Land cover for 27 vegetation classes in the study area was reclassified to 10 classes based on similarities between classes in the original landcover map and due to the importance of agricultural lands (5% of the study area) to onagers. Distance from agricultural lands, roads and villages were included as predictor variables, and were calculated with the Euclidean distance tool in the Spatial Analyst extension of ArcGIS 10.2. We checked for multi-collinearity among variables and correlation was <|0.7| between all pairs of variables. In addition, multi-collinearity among variables was further examined using USDM^31^ package version 3.5.3^24^ and variables with VIF (variance inflation factor) > 3 were used as a threshold to exclude variables^32^. VIF ranged from 1.2 to 1.8 for all variables. Therefore, all variables were retained for habitat modeling.

#### Resistance surface for connectivity analysis

To estimate landscape resistance, we converted the habitat suitability maps to resistance maps using a negative exponential function (R = 1000^(−1×HS)^) where R represents the cost resistance value assigned to each pixel and HS represents the predicted habitat suitability derived from the suitability models described above^33^. We used 1000 as the base of our exponential decay function such that areas with > 0.3 habitat suitability would have low-cost resistance. We rescaled the resistance values to a range between 1 and 100 by linear interpolation, such that minimum resistance (Rmin) was 1 when HS was 1, and maximum resistance (Rmax) was 100 when HS was 0^33^.

### Connectivity corridor network simulation

We used the universal corridor network simulator (UNICOR)^34^ to predict movement core areas and corridors for Onagers. UNICOR’s key features include a driver-module framework, connectivity mapping with thresholding and buffering, and graph theory metrics. UNICOR produces two kinds of connectivity predictions: (1) resistant kernels^16^ and (2) factorial least-cost paths^15^. The factorial least-cost path analysis implanted in UNICOR simulator uses Dijkstra’s algorithm^34^ to solve the single-source shortest path problem from every mapped species occurrence location on a landscape to every other occurrence location^34^. The analysis produces predicted least-cost path routes from each source point to each destination point. The resistant kernel algorithm calculates the resistance cost weighted dispersal kernel around each source point up to a user-defined dispersal threshold, and then sums these, producing an incidence function of the rate of organism movement through every pixel in the landscape as a function of the number and density of source points, the dispersal ability of the species, and the resistance of the landscape.

According to observation and reports of experts in the DoE, the maximum dispersal of threshold for movement of Onagers is about 100 km. We thus specified a dispersal threshold of 100,000 cost units for the resistant kernel analysis^35^. We calculated the factorial least-cost path network without dispersal the threshold^35^ to provide a broad-scale assessment of the regional pattern of potential linkage and to map corridors. The buffered least-cost paths were then combined through summation^15^ to produce maps of connectivity among all pairs of presence points.

### Evaluating congruence between crossing points and predicted connectivity

We used a spatial randomization testing procedure to evaluate congruence between the locations where onagers were observed crossing the road and resistant kernel values of predicted connectivity^18^. Spatial randomization testing of this kind is recommended in cases where there is spatial dependence among observations, and produces an unbiased estimate of the probability of the observed outcome given the data^18^.

We compared the median value of predicted connectivity (resistant kernel) for the 104 actual onager crossing locations with the distribution of median values of 1 × 10^7^ random samples of 104 locations along the highway within the study area. For each combination of resistance surface and connectivity modeling approach, we calculated the ranking of the median of observed values within the distribution of the medians of the 1 × 10^7^ random samples.

## Results

### Interviewees

Respondents were on average 45.5 years old (range = 20–80 years, median = 44 years SE = 0.26). 87% were farmers and ranchers, 6.3% were only farmers, 6.7% were ranchers. Most of the respondents (92.8%) owned agricultural lands, and 56.5% of their economic income was strongly dependent on agricultural lands. Local communities had an average of 2.5 ha of crop lands.

The majority of respondents had observed onager in the wild (88.5%), and 55% reported experiencing onager crop damage. Most people believed that the presence of onager around their village lead to crop damage (87.1%).

**Figure 2:**
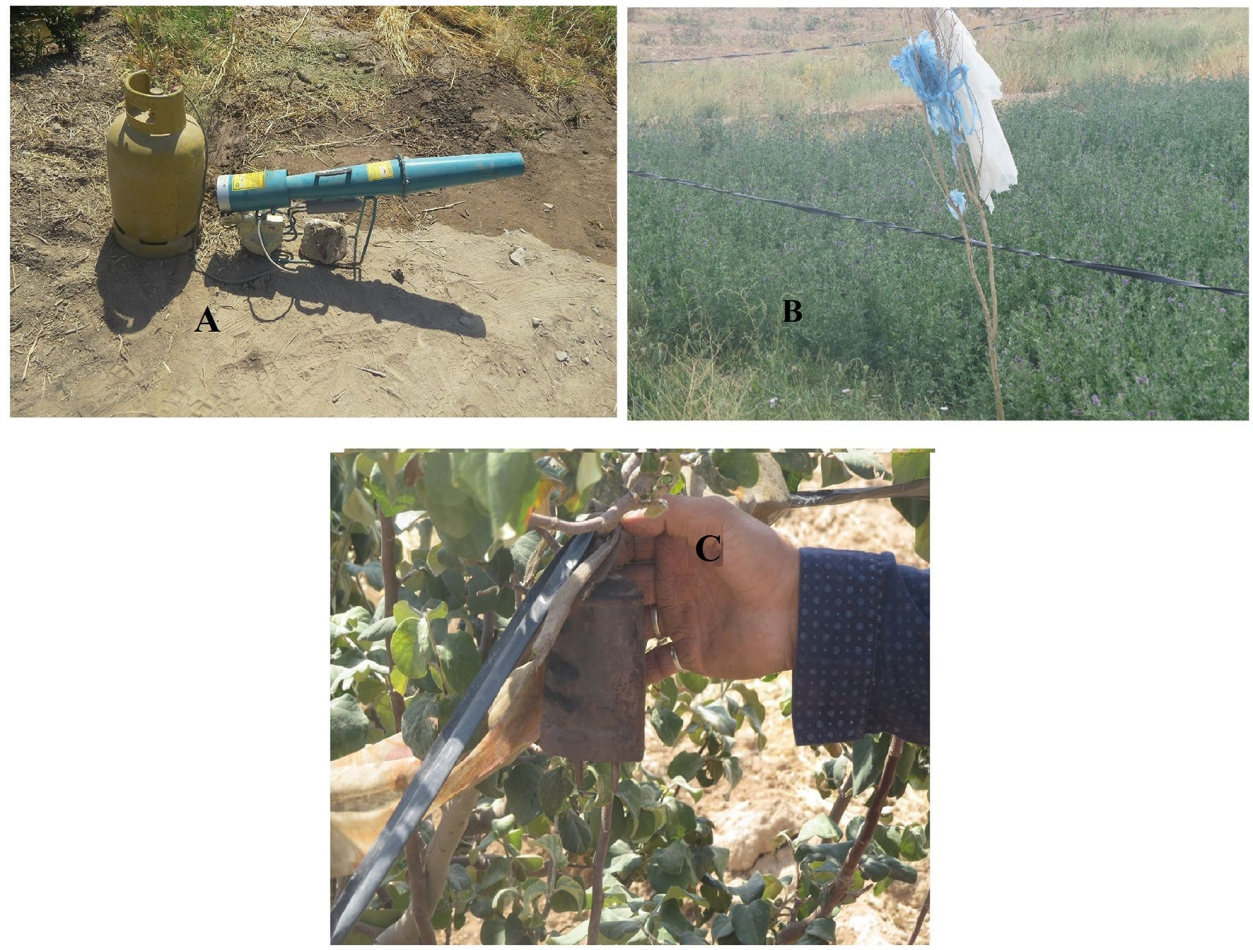
Traditional methods (**A**: Bird-Scarer (kalaghparan in Persian) **B**: Fencing use a plastic cuff and **C**: A plastic cuff with a bell on it) used by local communities for reducing HOC. Photo by Danial Nayeri and Alireza Mohammadi. Canon SX50 HS was used.

The majority of local people (59%) agreed with fencing around onager habitat, fencing their agricultural lands (95%), providing fodder and water for the onager (85.5%), buying fodder from local people by DoE (89.5%), and capturing and relocating the onager (71%). Most local people thought that guarding dogs (91%), scarecrow (99.5%), turning the lights on at night (97%) and deterrents such as terrifying sound machine and bird-scarers (57.5%; Fig. 2) were ineffective methods for mitigating onager crop damages. In contrast, fencing (96.5%) and using traditional barriers such as plastic cuff with a bell on it (61%) were considered effective methods to mitigate human-onager conflict.

Over half of the surveyed locals have experienced crop damage by onagers during the summer season (63%). Alfalfa (55%), barley (35%), and wheat (10%) were reported as the most susceptible crops to damage by onagers in this region. Most locals believed that drought and the lack of available forage in the wildlands are the main reasons for onagers approach the villages (98%). Only 2% of the local communities consider water scarcity as the main reason for the proximity of onagers to the villages.

We found that 25% of the respondents believe that onagers have no role in nature, and only 6% were knowledgeable of the ecological role of onagers and believed that the species disperses seeds and restores rangelands. Also, 7.5% of the respondents thought that onagers attracted tourists, 29.5% believe that Onagers are God's creation with intrinsic value, and 32% consider onagers to contribute to the beauty of nature.

Only 29.5% of the locals believe that onagers should be strictly conserved. 30% of the locals believe that the number of this species has increased but has caused tolerable damage with no significant conflict, and 36.5% of local people believe that their number has increased but also believe that decreasing onagers through hunting can mitigate human-onager conflict. Finally, 4% of local people believe that damages caused by onagers are intolerable and support immediate removal of the species from the region.

### Factors influencing Persian onager crop damages

Our omnibus tests of model coefficients were significant (χ^2 ^= 35.759, df = 16, P = 0.03), with goodness of fit = 140.109 for − 2 Log-likelihood, and Nagelkerke R^2^ = 0.280. Cox and Snell R^2^ was 0.164. The prediction accuracy of the model was 84.5%. In this analysis, the two significant variables were knowledge and local communities that used traditional methods for mitigating onager crop damages. We found that communities that were knowledgeable regarding the role of onagers (local people who believed: (1) by distributing seed of plants, the rangelands are restored, (2) onagers attract tourists in the region, and (3) onagers were part of the beauty of nature were less subject to crop damage by onagers (Table 2). Those who used traditional methods for mitigating onager crop damages (i.e., scarecrow and bird-scarer) were more prone to onager crop damage.

**Table 2:** Logit function coefficient of Factors influencing Persian Onager crop damages (Ln (p/(1−p) = 1.298 + 0.346 (X1) + 0.510 (X2) + 0.600 (X3) + 2.622 (X4) 1.876 (X5)).

Variable	B	S.E	Wald	df	Sig	Exp (B)	95% C.I.for EXP (B)	
							Lower	Upper
Do you Agree with Persian onager hunting? Yes	1.159	0.819	2.004	1	0.157	1.186	0.640	1.100
No	1.130	0.620	1.200	1	0.250	1.110	0.800	0.995
**Knowledge: What is the role of the Persian onager in the wild?**								
1: By distributing seed of plants, the rangelands are restored	0.346	0.110	6.210	1	0.012	1.230	1.070	2.010
2: It attracts tourists in the region	0.510	0.560	7.200	1	0.041	1.230	1.070	2.010
3: Beauty of nature	0.600	0.210	6.150	1	0.004	1.05	1.070	1.900
4: God’s creature with a right to life	0.065	.082	0.639	1	0.424	1.068	0.909	1.253
5: None	− 0.013	.237	0.003	1	0.957	0.987	0.621	1.570
**Which of these solutions could be effective in reducing Persian onager damages?**								
1: Fencing around the onager habitat	− 0.036	0.128	0.082	1	0.775	0.964	0.751	1.238
2: fencing around farmland	0.052	.0207	0.062	1	0.803	1.053	0.702	1.578
3: Give fodder and provide water for Persian onager	0.489	.0302	2.624	1	0.105	1.631	0.902	2.949
4: buying fodder from local people by DOE	− 0.787	.0538	2.140	1	0.143	0.455	0.159	1.306
5: capturing and relocating Persian onager	− 0.629	0.751	0.701	1	0.402	0.533	0.122	2.323
Education	− 0.009	0.210	0.002	1	0.964	0.991	0.656	1.495
Age	− 0.010	0.020	0.233	1	0.630	0.990	0.952	1.030
**Traditional Solution for reducing Persian onager damages:** 1: Guarding dogs	− .629	.751	.701	1	0.402	.533	.122	2.323
2: Fencing around agriculture land	1.280	1.657	4.322	1	0.384	0.635	0.289	1.821
3: Use of traditional barriers (a plastic cuff with a bell on it)	− 3.445	.912	0.756	1	0.238	0.032	0.001	0.821
4: Scarecrow	2.622	1.524	2.960	1	0.005	.073	.004	1.440
5: Turn on the lights at night	− 1.979	1.829	1.170	1	0.279	.138	.004	4.989
6: Bird-Scarer (kalaghparan In Persian)	1.876	1.117	2.817	1	0.009	6.524	.730	58.309
**Experience of Persian onager observation in nature**								
1 = Yes frequently	− 1.014	1.194	0.721	1	0.396	0.363	0.035	3.767
2 = Yes, several times	− 0.781	0.566	1.902	1	0.168	0.458	0.151	1.389
3: Yes, a few times	1.925	1.032	3.477	1	0.062	6.853	0.906	51.816
4: No, never	− 0.074	0.830	0.008	1	0.929	0.929	0.183	4.723
5: only seen the Persian onager carcass	− 0.070	0.282	0.061	1	0.805	0.933	0.536	1.622
**The presence of an Persian onager around your village damages your farms and gardens**								
1: Completely disagree	0.101	0.257	0.153	1	0.695	1.106	0.668	1.830
2: Somewhat disagree	0.173	0.401	0.187	1	0.665	1.189	0.542	2.609
3: I do not agree or disagree	− 0.008	0.217	0.001	1	0.972	.992	0.648	1.519
4: I agree somewhat	− 0.783	0.956	0.672	1	0.412	0.457	0.070	2.973
5: Completely agree	− 0.002	0.174	0.000	1	0.992	.998	0.710	1.403
Constant	1.298	1.750	0.550	1	0.458	0.273	–	–

### Classification of local communities by onager-caused crop damage

The Naïve Bayes algorithm was able to classify 192 out of 200 “No” cases (local communities who have no experience of crop-raiding damages) correctly and 188 out of 200 “Yes” cases (local communities have experience of crop-raiding damages) correctly. Thus the Naïve Bayes algorithm has 96% success in predicting the “No” group is about 96% and 94% success in predicting the “Yes” group.

**Figure 3:**
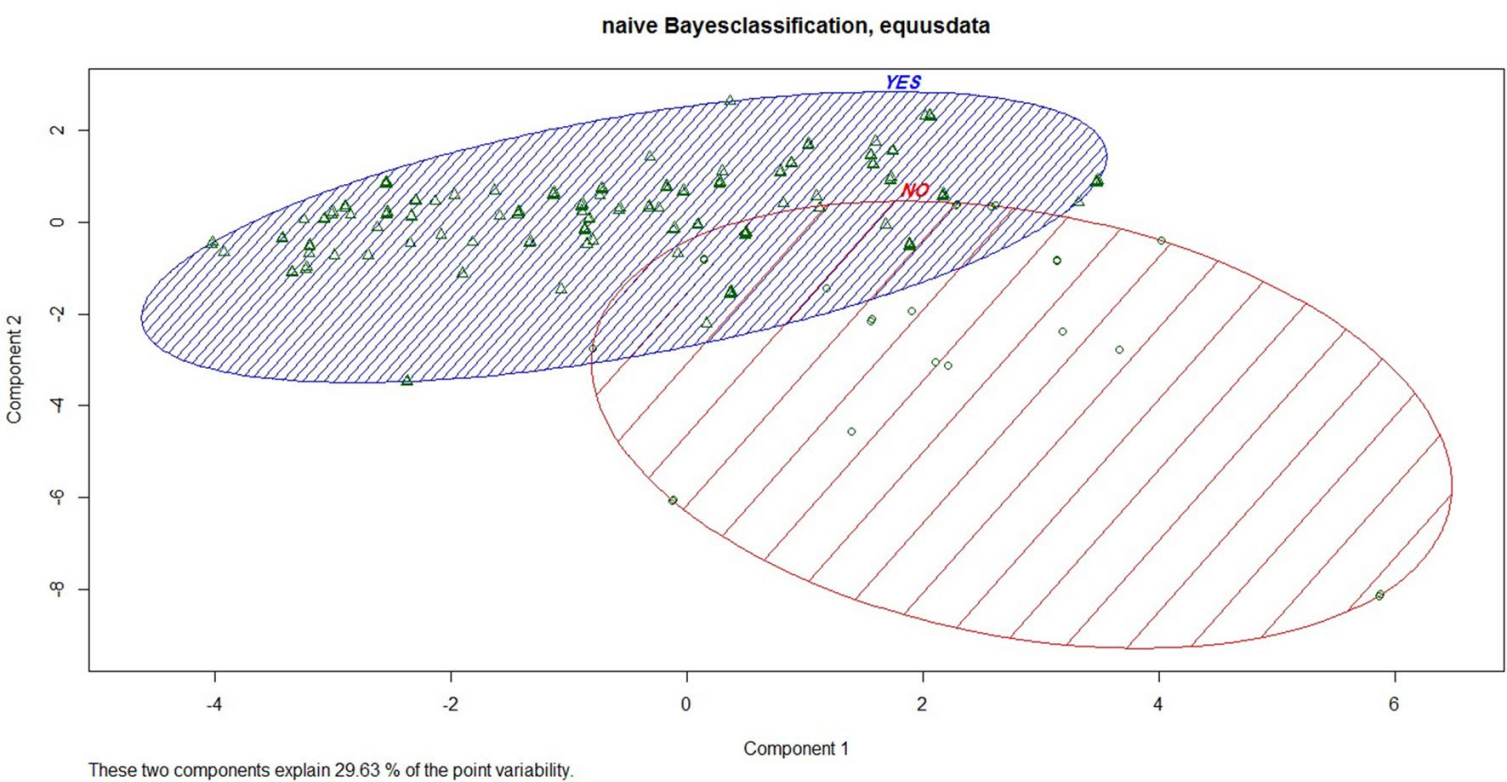
Classification of local communities using Naïve Bayes Classification according to damage or no damage of Persian Onager to agricultural products. In this figure the blue circle showed Yes group (local communities has experience of crop-raiding damages) and red circle showed No group (local communities who has no experience of crop-raiding damages). Triangles showed distribution of individual in Yes group and circles showed distribution of individual in No group.

Our results showed that the most significant variables for predicting onager crop-raiding were farmland area, the opinion of onager as a serious threat to agricultural lands, and local communities who used traditional methods. Our model supported that: (1) local respondents with more agricultural lands were more subject to onager damage, (2) local respondents who agree that onagers are a significant threat are more subject to crop damage, and (3) local respondents who use traditional methods such as scarecrow and bird-scarer to exclude onagers were more subject to onager damages (Fig. 3).

### Habitat conectivity

**Figure 4:**
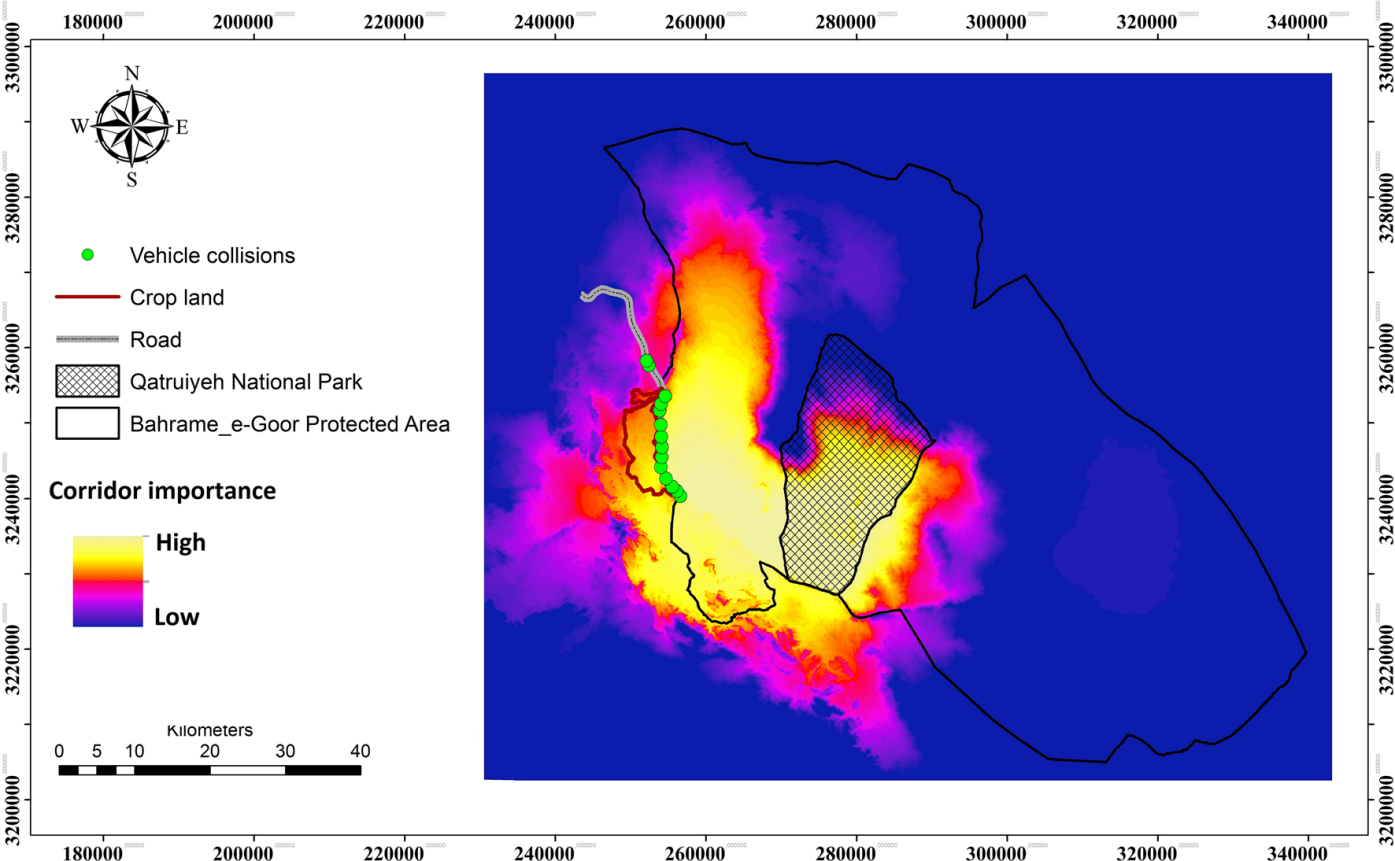
Resistant kernel core habitat areas (bright spot) for The Persian Onager in Bahram-e- Goor Protected Area in Iran. Connectivity models revealed that core areas of movement are highly concentrated at the center of protected areas. UNICOR (https://www.fs.usda.gov/treesearch/pubs/40686) software was used to generate the figure.

**Figure 5:**
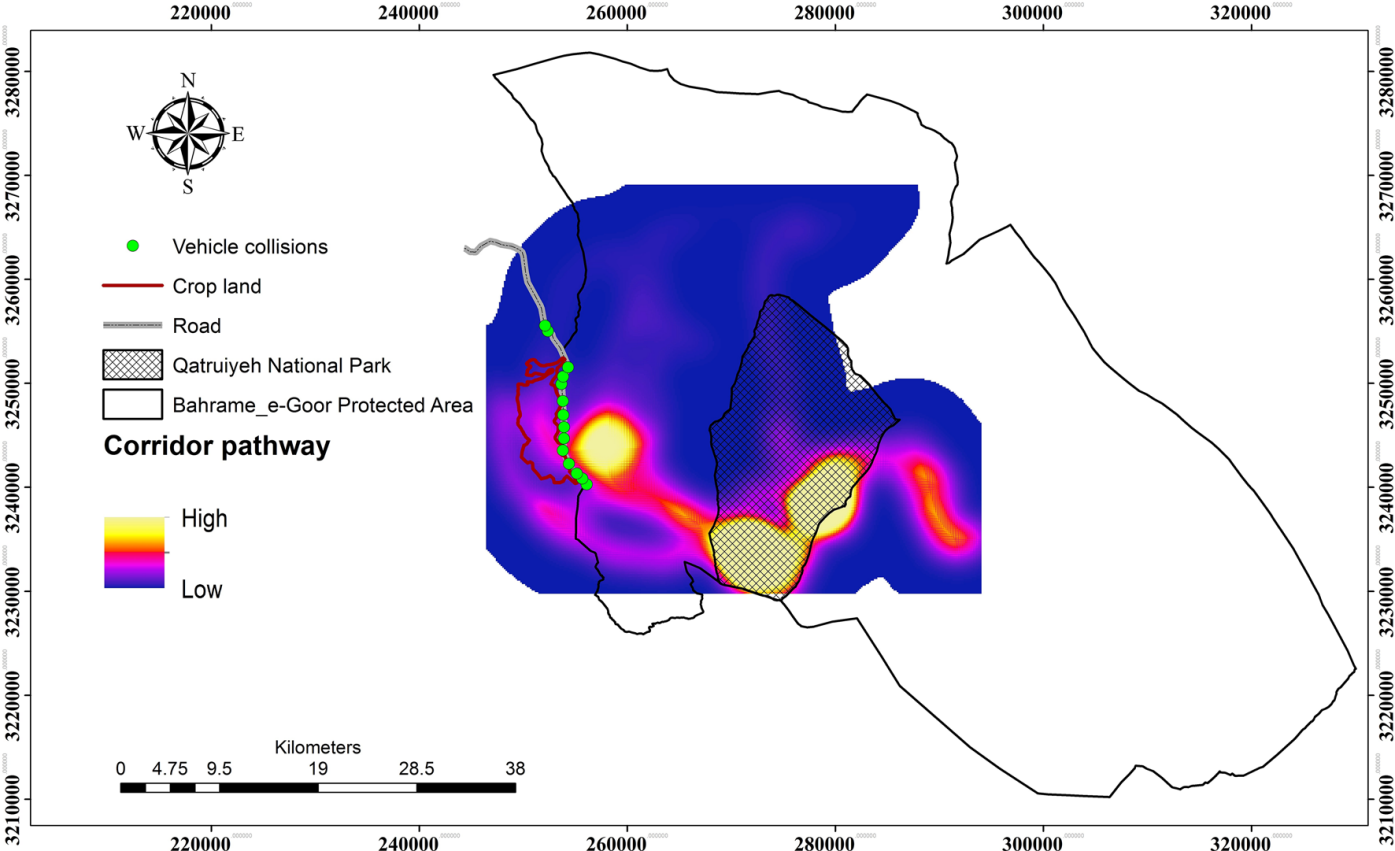
UNICOR corridor pathways for The Persian Onager in Bahram-e- Goor Protected Area in Iran. UNICOR (https://www.fs.usda.gov/treesearch/pubs/40686) software was used to generate the figure.

Our MaxEnt model predictig Onager occurrence locations was highly predictive (AUC = 0.89). Our connectivity simulation modeling revealed that high connectivity areas are in the southern parts of the Qatrouyeh National Park and in the western parts of the Bahram–e-Goor Protected Area. Also, connectivity extends through the west side of the protected area (Figs. 4 and 5).

### Spatial randomization test

We found that 14 onagers were killed on the Hassan Abad-Meshkaan road during 2004–2018 (Table 3). Most onager road kills occurred in the summer season (Table 3). Most of the onagers that collided with vehicles were female (Table 3).

**Table 3:** Road mortalities of the Persian onager during 2004–2018.

Year	Sex	Season
2004	Female	April
2004	Unknown	August
2006	Male	August
2007	Female	August
2008	Male	August
2009	Male	August
2011	Female	August
2017	Female	December
2017	Female	December
2017	Female	August
2017	Male	April
2017	Male	May
2018	Female (Pregnant)	April
2018	Female (Pregnant)	April

**Figure 6:**
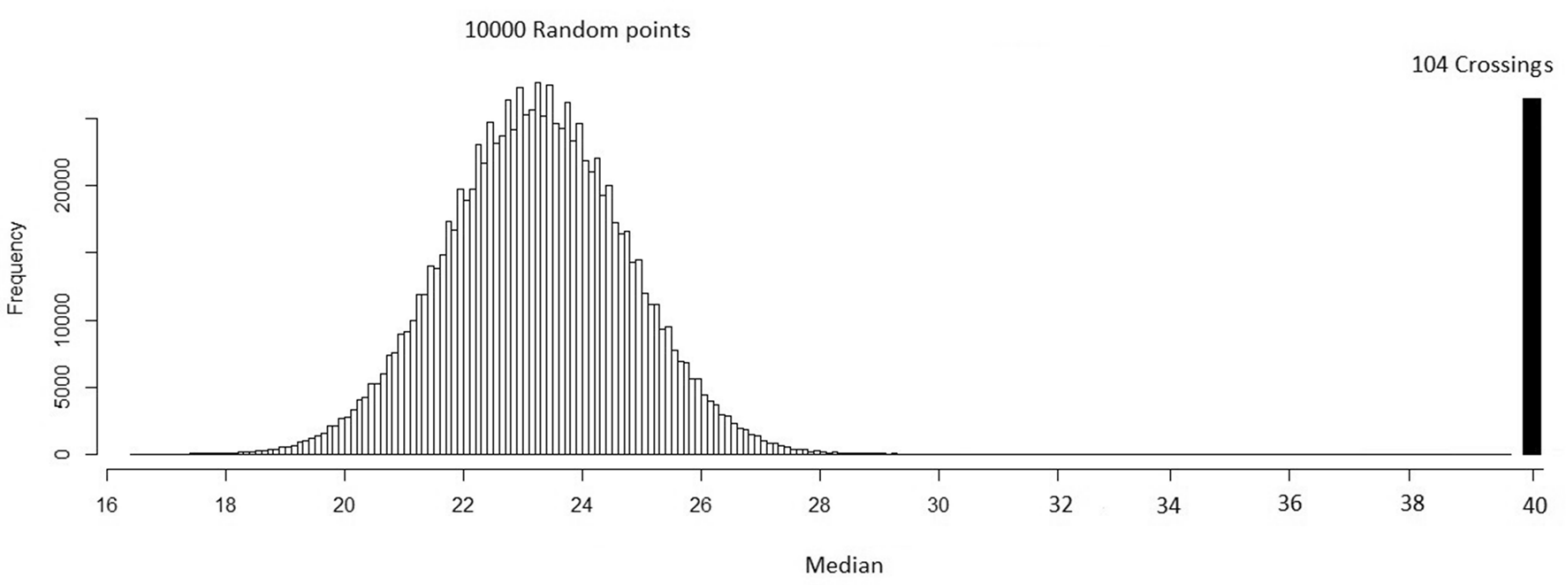
Spatial randomization test: the crossings have a much higher connectivity score than the randomization. Solid vertical line shows the median of 104 crossing locations. Tiny bars shows 10,000 random points. This means that connectivity model predicted Onager highway crossing locations precisely.

Our connectivity model strongly predicted onager highway crossing locations. Specifically, crossing locations have a much higher connectivity score than the distribution of randomized locations (Fig. 6). The median predicted connectivity value of 10,000 random points on the road was 22, compared to a median connectivity value of 40 for the 104 onager crossing points considered in our analysis. The highest observed connectivity value of actual crossing points (104 points) was 49, which is higher than the highest value among the random points (10,000), which was 30. Based on the randomization test the probability of the observed value being no different than the random value is < 0.00001.

## Discussion

Studies combining social sciences with habitat connectivity are scant globally^36^. Moreover, there has been little research about human-onager conflict in the study area^37^ despite its holding one of the largest remaining populations of Persian onager and also being an area of particularly high human-onager conflict. Hence, our study is one of the first studies that integrated spatial ecology of an endangered species with its human dimensions to facilitate sustainable coexistence. Furthermore, it is a fundamental first step to understanding the sociological and ecological drivers of conflict and identifying solutions to mitigate it. In general, we believe there is high value in assessing the knowledge and attitudes of the local people regarding onager, crop damage, and the best ways to mitigate it. Understanding the nature of human-wildlife conflict, and seeking means to resolve it, requires quantitative information about both the attitudes of people and the ecology and behavior of the wildlife species involved^38^.Understanding that attitudes of local people coexisting with onagers is essential to effectively solve conflict problems. In addition, knowing the traditional methods used by local people to mitigate conflict and their applicability can aid managers to develop cost-effective solutions.

### Minimizing onager crop-raiding

Food availability is a critical factor in onager conflict risk (Table 4). Scarcity of natural forage during drought conditions leads to onagers crossing roads and entering agricultural fields^39^ (Table 4). In similar systems, crop-raiding conflicts involving the khur subspecies (*E. h. khur*), for example, have recently increased because of natural dispersal of increasing populations into adjacent agricultural landscapes^10^. In contrast to findings from Esmaeili et al. (2019)^37^, human-wildlife conflicts in our study area are relatively high (in comparison to past years), especially during dry summers and cold winters when forage is scarce. At such times onagers are forced to leave the protected areas and enter neighboring agricultural lands looking for food. Our results show that crop-raids by onagers and vehicle collisions both increase during these times (Table 4). Fences for excluding onagers from crops have been proposed as a solution. However, installing fences that can cover the entire study area is costly and impractical without financial subsidies from the government.

**Table 4:** Driving forces, threats and recommendations for human-onager conflicts in southern Iran.

Driving forces	Threats	Recommendations
Reduced food availability and water during drought season	Increasing conflicts and road vehicle collisions	(1) Increasing public awareness and educational programs (2) Building artificial water ponds and supplying hay in areas away from Agricultural lands (3) Insurance scheme for agricultural lands (4) Improving road safety for drivers and onagers

Increasing public awareness and educational programs may be a potential solution to human-onager conflicts. Our study shows that local respondents who are aware of the issues around onagers are less subject to crop damages compared with those who only use traditional tactics (e.g., scarecrow, guarding dogs, turn on lights at night and other deterrents) for mitigating crop damages. Our results confirmed our first hypothesis that people who are knowledgeable of the ecological role onager play in the ecosystem are less likely to experience crop damage. Our study is not able to separate causes versus correlations regarding attitudes of people toward onager. However, our results suggest that increasing the ecological knowledge of the local people about this species can help to promote co-existence and potentially reduce onager-crop damage. Most of the local people have a respectful attitude toward onagers and consider them as a natural heritage of their homeland, leading to support for onager conservation^40^.

Conservation actions such as building artificial water ponds and supplying hay in areas away from agricultural lands may reduce chances of onagers from entering local farms^4^. These approaches are likely to be more financially feasible than installing fences over a large area. We also suggest that future studies examine alternative livelihoods and modifying crop cultivation for reducing human-wildlife interaction in this area.

### Minimizing onager vehicle collisions

While crop-raiding is a primary nexus in human-onager conflict, the major threat for safe movement of onagers within their habitats is road crossing east–west through the landscape. Our corridor modeling demonstrated that the road on the western border of Bahram–e-Goor Protected Area had high impacts on onager movement, and this area has a concentration of onager-vehicle collisions. Roads were predicted to substantially reduce connectivity of onagers, which confirms our second hypothesis. Further, a large portion of onager mortality in the study region was related to road kills. Previous studies also suggest that roads are a predominant threat to animals due to road collisions and fragmentation of habitat^41,42^.

Mitigation measures to safeguard movement and road crossing of onager individuals should be considered in this area, particularly along the corridor routes we identified and validated with spatial randomization testing (Table 4). Our analysis confirmed that actual onager crossing locations were highly nonrandom, and closely associated with our predicted corridor locations. This is similar to the results of Cushman et al.^18^ for American black bear, and of Mohammadi et al. (in press) for grey wolf and jackal in Iran, suggesting that synoptic connectivity methods, such as resistant kernel and factorial least cost paths, are effective in predicting and prioritizing highway locations for mitigation^43^.

Although some parts of the border of the protected area were fenced along the road (south of the Bahram-e-Goor Protected Area), some parts of the fence have been destroyed, and onagers cross the road and enter the villages at night and damage crops. Such movements not only intensify the risk of vehicle collisions but also generate problems for local livelihoods by raiding crops and trampling. We thus suggest rebuilding and expanding the fences in strategic locations that provide a barrier between onager habitat and crops, or in places that actively funnel movement of onagers between habitat patches and around agricultural areas. Provision of more waterholes in the outlying areas may also mitigate the need for onagers to visit village ponds or irrigation tanks, and may help keep onagers away from croplands.

The DoE has designed and prepared eight onager warning signs, for increasing public awareness. We recommend installing these signs at the crossing locations highlighted in our connectivity analysis. Persian onager mortality rates and critical locations should be assessed before installation of the signs to help optimally locate them and enable subsequent measurement of their effectiveness in the reduction of onager collision. Most studies on the effectiveness of signs in reducing WVCs could not ensure their effectiveness^42^. Additionally, the previous design of the warning signs for Persian onager did not meet the conventional standards of size, color, direction, and distance to the road. Previously installed warning signs are barely perceptible at night. We propose to install warning lights at the top of each onager warning sign, and to locate the signs at specific locations where crossing was accurately predicted in our models and where high rates of crossing and road kill have been documented.

We also suggest holding educational workshops to increase awareness in parallel to other conservation initiatives. The area has the potential to support tourism for onager watching, which can also bring economic incentives for local communities. We encourage the promotion of ecotourism in our study area. By emphasizing the tourism value of the rural landscapes it can attract visitors from wealthy urban regions and promote awareness and appreciation of conserving the traditional landscapes and wildlife within while bringing in external income to support conservation goals.

We recommend experimenting the involving of local communities in onager monitoring efforts through financial incentives, which may help to track and pinpoint hotspots where conflicts are mostly likely to occur. This would provide information for mitigating road mortality and crop-raids. The key to sustainable onager conservation in wild land-pastoral-agricultural interface requires community-based initiatives. There is a critical need to improve the understanding and appreciation of local communities towards biodiversity in general, and towards onagers in particular, for better ways to manage agricultural activities that can reduce onager damage.

## Conclusions

Human-onager conflict is currently characterized by two important factors: (1) crop-raiding during drought seasons and (2) vehicle collisions. The first step toward implementing mitigation strategies is helping local people protect their lands and initiating compensation through insurance schemes. Another step is to improve road safety for drivers and onagers. Our analysis identified the key habitat, core and movement areas for onagers, and demonstrated that our connectivity analysis predicted highway crossings with great accuracy. Management interventions such as building artificial water ponds, fencing and supplying hay during drought season could also be implemented to reduce onagers entering local’s farms. In addition, encouraging ecotourism and educational workshops could help improve understanding and tolerance of onager in the community.

## Supplementary Information

Supplementary Information.
