# Supplementary material for: Integrating spatial analysis and questionnaire survey to better understand human-onager conflict in Southern Iran
Source: Sci Rep. 2021 Jun 14;11:12423. doi: 10.1038/s41598-021-91921-w (PMC8203636; doi:10.1038/s41598-021-91921-w)
Supplement: Supplementary file 1 — Supplementary Information. [file 41598_2021_91921_MOESM1_ESM.pdf]

Integrating Spatial Analysis and Questionnaire Survey to Better Understand Human-Onager Conflict in Southern Iran

Authors

Alireza Mohammadi[1*], Kamran Almasieh[2], Ho Yi Wan[3], Danial Nayeri[4], Amir Alambeigi[5], Jason I. Ransom[6], Samuel A. Cushman[7]

1. Department of Environment Sciences and Engineering, Faculty of Natural Resources, University of Jiroft, Jiroft, Iran.
2. Department of Nature Engineering, Agricultural Sciences and Natural Resources University of Khuzestan, Mollasani, Iran.
3. Department of Wildlife, Humboldt State University, 1 Harpst Street, Arcata, CA 95521, United States.
4. Department of Environmental Sciences, Faculty of Natural Resources, University of Tehran, Karaj, Iran.
5. Department of Agricultural Extension and Education, College of Agricultural Economics and Development, University of Tehran, Karaj, Iran.
6. Department of Ecosystem Science and Sustainability, Colorado State University, Fort Collins, Colorado, USA.
7. USDA Forest Service Rocky Mountain Research Station, 2500 S. Pine Knoll, Flagstaff, AZ, USA.

[*]Corresponding author: Alireza Mohammadi

Email: armohammadi1989@gmail.com

Fax: (034)-43347065   Phone: (034)-43347061

Supplementary materials

Figures:

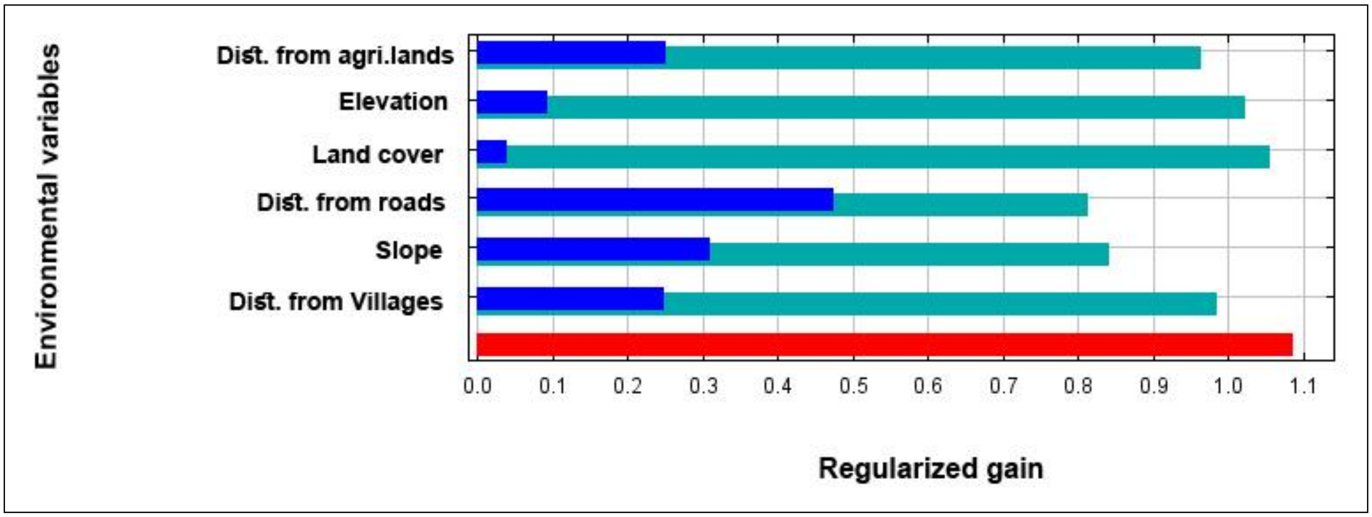

Fig. S1. Jackknife Analysis within MaxEnt to determine the importance of each environmental layer in habitat suitability modeling of the onager in the study area.

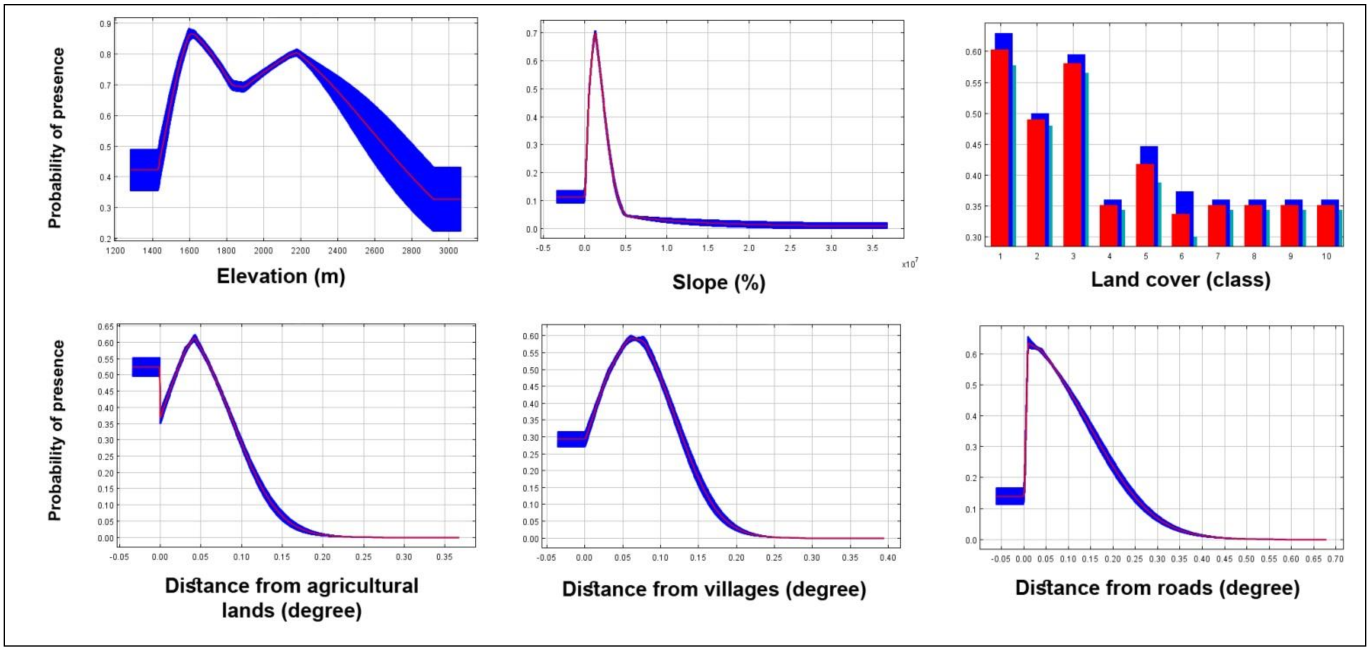

Fig. S2. Response curves of the onager presence to environmental layers in the MaxEnt model, Numbers on X-axis of land cover curve represent: 1) agricultural lands, 2) low-density rangelands 3) moderate-density rangelands, 4) human settlements, 5) low-density forests, 6) bare lands, 7) salt lands, 8) rocks and 9) wetlands.

Tables:

Table S1. Descriptive statistics (percent) for the items used in this study (sample size, n=200). Items are codified according to the instruments section (see text for details).

Instrument	Items (Percent)
**Solution for reducing Persian onager damages:**	
1: Fencing around the onager habitat	1: Yes (41)
2: fencing around farmland	2: Yes (5)
3: Give fodder and provide water for Persian onager	3: Yes (14.5)
4: buying fodder from local people by DOE	4: Yes (10.5)
5: capturing and relocating Persian onager	5: Yes (29)
**Traditional Solution for reducing Persian onager damages:**	
1: guarding dogs	1: Yes (9)
2: fencing around agriculture land	2: Yes (96.5)
3: Use of traditional barriers (a plastic cuff with a bell on it)	3: Yes (61)
4: Scarecrow	4: Yes (0.5)
5: Turn on the lights at night	5: Yes (3)
6: Bird Scarer (kalaghparan in Persian)	6: Yes (57.5)
**Personal experience**<br>1: Have you ever seen a Persian onager in the wild?	1= Yes frequently (88.5)<br>2= Yes, several times (10)<br>3: Yes, a few times (0.5)<br>4: No, never (0.5)<br>5: only seen the Persian onager carcass (22.5)

2: Have you had any of your crops damages by a Persian onager during the last year?	2: Yes (88)
3: The presence of a Persian onager around your village damages your farms and gardens. How much do you agree with this statement	1: completely disagree (3)<br>2: Somewhat disagree (0)<br>3: I do not agree or disagree (1)<br>4: I agree somewhat (8.5)<br>5: completely agree (87.1).
**Knowledge:**<br>What is the role of the Persian onager in the wild?	1: By distributing seed of plants, the rangelands are restored<br><br>2: It attracts tourists in the region<br><br>3: Beauty of nature<br><br>4: God’s creature with a right to life.<br><br>5: None
What season is the most time you see a Persian onager in nature?	1: spring<br>2: summer<br>3: autumn<br>4: winter
In your opinion, what factors, you think, causes Persian onager to move in human-dominated landscape?	1: drought (lack of forage)<br><br>2: lack of water
**Opinion:**<br>How do you assess Persian onager situation	1: A species needs to be strictly conserved<br>2: Their number has increased but causes tolerable damage and causes no conflict<br>3: Their number has increased but with decreasing it through hunting can mitigate HOC<br>4: The damage is intolerable and species need to be removed from the area immediately
If the conflict is increasing in the area, who is primarily responsible?	1: Increase in Persian onager population

	2: Department Of Environment (DOE)<br>3: crop pattern<br>4: Decrease the tolerance level of local people<br>5: drought (lack of forage)
What do you think the most important reason for the threat of a Persian onager in the region?	1: hunt<br>2: vehicle collision<br>3: Habitat fragmentation<br>4: drought (lack of forage)
Do you want to help DOE for conserving this species?	1= Yes, 0= No
**Do you agree with Persian onager hunting?**	1= Yes, 0= No
**Interest:**<br>Are you interested in Persian onager	1: completely like (3)<br>2: Somewhat like (0)<br>3: Indifferent (1)<br>4: I don’t like (8.5)
**Job**<br>2; Farmer<br>3: Shepherd,<br>4: Agriculture worker<br>5: Free job<br>6: Rancher and Farmer	1: Rancher (1.5)<br>2: Farmer (49)<br>3: Shepherd (1.5)<br>4: Agriculture worker (1)<br>5: Free job (13)<br>6: Rancher and Farmer (34%)
**Age**	1: <30 years (8)<br>2: 30-50 Years (50)<br>3: 50 Years (42)
**Education**	1: Incomplete Elementary lower than 5th grade of elementary (31)<br>2: Complete Elementary 5th grade of elementary (16.5)<br>3: Incomplete High school (36)<br>4: Associate Degree (10)<br>5: Bachelor of Science (BSc) (2)<br>6: Master of Science (MSc) or Higher (4.5)
